# Metagenomic characterization of fire-adapted soil microbiomes in the Albany Pine Bush Preserve

**DOI:** 10.1128/mra.00664-25

**Published:** 2025-08-27

**Authors:** Alaina O. Benot, Gray Waldschmidt, Chanistha Tiyapun, Isaac J. Okyere, Jennifer L. Goff

**Affiliations:** 1 Department of Environmental Biology, SUNY College of Environmental Science and Forestry 14797, Syracuse, New York, USA; 2 Department of Sustainable Resources Management, SUNY College of Environmental Science and Forestry 14797, Syracuse, New York, USA; 3 Department of Chemistry, SUNY College of Environmental Science and Forestry 14797, Syracuse, New York, USA; Montana State University, Bozeman, Montana, USA

**Keywords:** fire, forest, pine bush, environmental microbiology, soil microbiology, metagenomics, microbial ecology

## Abstract

The Albany Pine Bush Preserve’s documented fire history enables a unique study of fire-dependent ecosystems. We identified 94 unique bacterial and archaeal metagenome-assembled genomes spanning 27 classes, providing genomic insights into microbial nutrient cycling in these systems.

## ANNOUNCEMENT

The Albany Pine Bush Preserve (APBP) (Albany, NY, USA; 42.719°N, 73.864°W) is one of the largest inland pine barrens in North America and is notable for its extensive fire management history. As a fire-dependent ecosystem, the APBP relies on prescribed burns to prevent succession to closed-canopy hardwood forests, preserving this rare ecosystem and its unique biodiversity (1). Soil microbiota drive the nutrient cycling that supports a healthy pine barren ecosystem (2). Thus, characterization of soil microbiomes across a gradient of fire history in the APBP uncovers how fire shapes the microbial community structure and function and, ultimately, this endangered ecosystem.

Sampling plots in the APBP were chosen by frequency of fire return: “frequent” experienced ≥4 scorching fires in the last 30 years (*n* = 3), “infrequent” experienced 2–3 in the last 30 years (*n* = 3), and the “control” had no fire history since 1995 (*n* = 3). Within these nine plots, four locations were chosen for sub-sampling at random. From each sub-sample location, we extracted two soil depths (0–15 and 15–30 cm). To capture the soil heterogeneity in our metagenomes, 100 g from each sub-sample at both depths was homogenized—yielding 18 homogenized samples for sequencing—and stored at −80°C until analysis.

We extracted DNA from 250 mg of the homogenized samples using the ZymoBIOMICS DNA Miniprep Kit (Lot no. 251052), following the manufacturer’s instructions. DNA sequencing and library preparation were performed at the University of Delaware Center for Bioinformatics and Computational Biology. Library preparation was performed with the Illumina DNA preparation kit, following the manufacturer’s instructions. Short-read sequencing was performed on an Element AVITI using Cloudbreak Freestyle chemistry. On average, each sample (*n* = 18) generated 248,341,896 paired-end, 150 bp reads (range 234,028,462 to 268,953,762; a detailed table is uploaded at https://doi.org/10.6084/m9.figshare.29594432.v1).

Our metagenomic workflow was carried out using the US Department of Energy’s Knowledge Base (KBase) platform (3) with default settings for each application, unless otherwise specified. Trimmomatic v0.39 (4) processed read trimming and quality control of the raw reads using adaptor clipping option for Nextera PE-PE adaptors. Reads were assembled using MetaSPAdes v3.15.3 (5). Binning was performed using MetaBAT2 v1.7 (6), CONCOCT v1.1 (7), and MaxBin2 v2.2.4 (8) with markers 40 for bacterial and archaeal genes and 107 for bacterial genes. DasTOOL v1.1.2 (9) refined all bins for consensus. Resulting bins were filtered for quality at 50% completion rate with 10% contamination via CheckM v1.0.18 (10) and were annotated using RASTtk v1.073 (11, 12). We performed taxonomic identification using GTDB Toolkit v2.3.2 (13).

**Fig 1:**
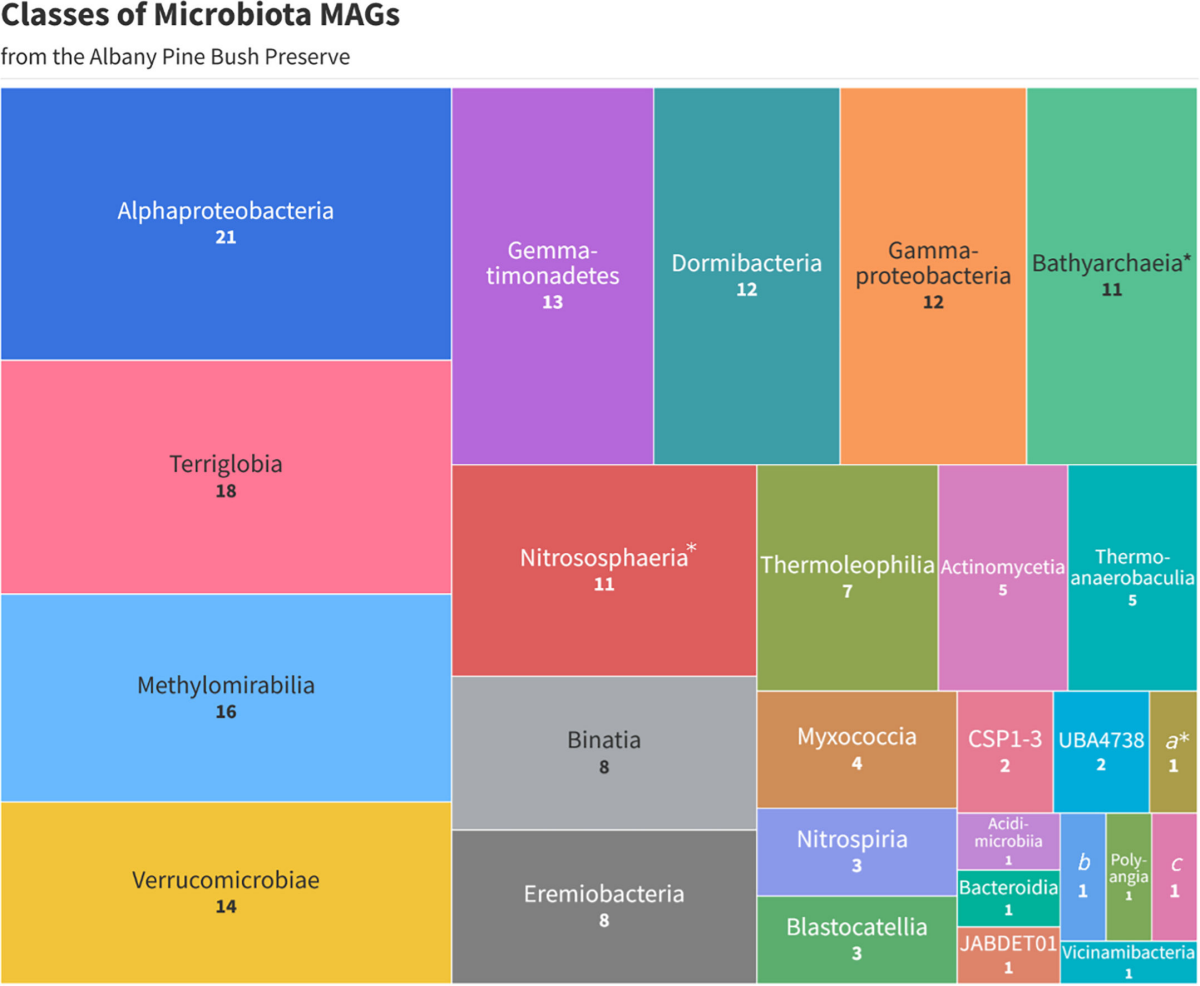
Treemap displaying the 27 distinct classes extracted from the microbiota MAGs of the Albany Pine Bush Preserve. Classes with (*) indicate archaea. Classes a*, b, and c have names that are too long to display properly: a*, Thermoplasmata, b, Limnocylindria, and c, Saccharimonadia.

Following these steps, the resulting 183 bins were dereplicated at 95% ANI, calculated with FastANI v0.1.3 (14), choosing the highest quality MAG as the representative for each cluster of genomes. This yielded 94 distinct metagenome-assembled genomes (MAGs) representing 27 classes (3 archaeal, 24 bacterial), as shown in Fig. 1 (a detailed table is uploaded at https://doi.org/10.6084/m9.figshare.29176256.v2). On average, completion and contamination rates were 80.43% and 3.20%, respectively, with an average GC content of 61.60%. Each MAG had an average of 355 contigs and an average total length of 3.4 million base pairs. MAGs derived from APBP soil will provide new insights into fire-dependent ecosystems and their associated biogeochemical cycles.

## Data Availability

All data are associated with BioProject PRJNA1269515. Raw sequencing data are available through NCBI Sequence Read Archive (SRA) via accession numbers SRX28992350–SRX28992355; SRX29211976–SRX29211981; and SRX29333943–SRX29333948. MAGs are publicly available through Figshare at https://doi.org/10.6084/m9.figshare.29424812.v4 alongside file information at https://doi.org/10.6084/m9.figshare.29424809.v1. MAG assembly statistics, taxonomy, and metadata are publicly available through Figshare at https://doi.org/10.6084/m9.figshare.29176256.v2. Metagenome read library information and metadata are publicly available through Figshare at https://doi.org/10.6084/m9.figshare.29594432.v1.
